# CRISPR/Cas9-mediated editing of double loci of *BnFAD2* increased the seed oleic acid content of rapeseed (*Brassica napus* L.)

**DOI:** 10.3389/fpls.2022.1034215

**Published:** 2022-11-22

**Authors:** Han Liu, Baogang Lin, Yun Ren, Pengfei Hao, Lan Huang, Bowen Xue, Lixi Jiang, Yang Zhu, Shuijin Hua

**Affiliations:** ^1^ Institute of Crops and Nuclear Technology Utilization, Zhejiang Academy of Agricultural Science, Hangzhou, China; ^2^ Department of Seed Management, Yongding Agriculture and Rural Bureau of Longyan, Longyan, China; ^3^ Huzhou Agricultural Science and Technology Development Center, Institution of Crop Science, Huzhou, China; ^4^ College of Agriculture and Biotechnology, Zhejiang University, Hangzhou, China

**Keywords:** CRISPR/Cas9, genome editing, mutation, oleic acid, rapeseed, sequencing, yield

## Abstract

Seed oleic acid is an important quality trait sought in rapeseed breeding programs. Many methods exist to increase seed oleic acid content, such as the CRISPR/Cas9-mediated genome editing system, yet there is no report on seed oleic acid content improvement *via* this system’s precise editing of the double loci of *BnFAD2*. Here, a precise CRISPR/Cas9-mediated genome editing of the encoded double loci (A5 and C5) of *BnFAD2* was established. The results demonstrated high efficiency of regeneration and transformation, with the rapeseed genotype screened in ratios of 20.18% and 85.46%, respectively. The total editing efficiency was 64.35%, whereas the single locus- and double locus-edited ratios were 21.58% and 78.42%, respectively. The relative proportion of oleic acid with other fatty acids in seed oil of mutants was significantly higher for those that underwent the editing on A5 copy than that on C5 copy, but it was still less than 80%. For double locus-edited mutants, their relative proportion of oleic acid was more than 85% in the T_1_ and T_4_ generations. A comparison of the sequences between the double locus-edited mutants and reference showed that no transgenic border sequences were detected from the transformed vector. Analysis of the *BnFAD2* sequence on A5 and C5 at the mutated locus of double loci mutants uncovered evidence for base deletion and insertion, and combination. Further, no editing issue of *FAD2* on the copy of A1 was detected on the three targeted editing regions. Seed yield, yield component, oil content, and relative proportion of oleic acid between one selected double loci-edited mutant and wild type were also compared. These results showed that although the number of siliques per plant of the wild type was significantly higher than those of the mutant, the differences in seed yield and oil content were not significant between them, albeit with the mutant having a markedly higher relative proportion of oleic acid. Altogether, our results confirmed that the established CRISPR/Cas9-mediated genome editing of double loci (A5 and C5) of the *BnFAD2* can precisely edit the targeted genes, thereby enhancing the seed oleic acid content to a far greater extent than can a single locus-editing system.

## Introduction

Rapeseed (*Brassica napus* L.) is the next most important oil crop after soybean (*Glycine max*) in terms of both the land area under cultivation and seed yield worldwide. Rapeseed oil that is low in both erucic acid and glucosinolate is considered high-quality edible oil due to its high percentage (>90%) of C18 unsaturated fatty acids and other healthy secondary metabolites, such as vitamin E, sterol, and polyphenols (Barth, 2009; Chew, 2020; Tan et al., 2022). Among these unsaturated fatty acids, oleic acid, linoleic acid, and linolenic acid are the major constituents of seed oil. Oleic acid is recognized as a type of healthy fatty acid; for example, it can reduce blood pressure by regulating the membrane lipid structure to control G protein-mediated signaling (Terés et al., 2007; Terés et al., 2008). Furthermore, a higher oleic acid content of rapeseed oil can prolong its shelf time because of a lower ratio of oxidation in comparison with conventional oil having a low oleic acid and high linolenic acid content (Roszkowska et al., 2015; Cao et al., 2020; López et al., 2022). For example, Merrill et al. (2008) found that the oil stability index of high-oleic canola, high-oleic sunflower, and very high-oleic canola was higher than that of sunflower, soybean, canola, corn, partially hydrogenated soybean, and oleic safflower. Normally, oleic acid contributes about 55% to 65% of total fatty acids in most modern rapeseed varieties used for cultivation (Hu et al., 2006). Rapeseed has a much lower oleic acid percentage than oil crops such as soybean, sunflower, and peanut (Dhakal et al., 2014; Alberio et al., 2016; Konuskan et al., 2019). Compared with other oil crops, the advantages of rapeseed oil with high oleic acid percentage are as follows: firstly, its larger cultivation area and higher oil yield can offset the shortage due to the low oil yield of other oil crops; secondly, its higher percentage of the high level of oleic acid that is a monounsaturated fatty acid relative to polyunsaturated fatty acid, linoleic acid, and linolenic acid would make it healthier. Therefore, increasing the amount of seed oleic acid in rapeseed is an imperative breeding objective from the nutrition perspective.

Understanding the genetic control of oleic acid content is crucial to the breeding program for high oleic acid improvement in rapeseed. Previous investigations suggested that rapeseed had high heritabilities (*h* ^2^ = 0.94) for the seed oleic acid content within both high and low oleic acid types (Schierholt and Becker, 2001). This suggests that the seed oleic acid of rapeseed might be controlled by a handful of genes or some major effective loci (Velasco et al., 2003), which also suggests the strong possibility to breed high oleic acid in rapeseed *via* biotechnological manipulation of just a few genes or loci. One attempted way to breed a high oleic acid variety relied on ethyl methanesulfonate (EMS) mutation (Spasibionek, 2006; Lee et al., 2018). The seed oleic acid content can reportedly reach at least 85% in the F_4_ generation after EMS mutagenesis; however, low amounts of seed were also observed despite abundant pollen, suggesting that fertility might be impaired by this method (Auld et al., 1992).

Because the EMS mutagenesis method depends on a large population for targeted trait screening, leading to laborious work for researchers, other methods are sought to be explored. Genetic engineering achieved *via* RNA interference (RNAi) knockdown and genome editing of critical genes controlling seed oleic acid content, to augment the seed oleic acid content, could offer an effective way forward. The *FATTY ACID ELONGASE* (*FAE*) gene is responsible for erucic acid content in rapeseed (Kanrar et al., 2006; Wu et al., 2015). Previous investigations demonstrated that fatty acid elongation mutants in rapeseed and *Arabidopsis* affected elongation from C_18:1_ to C_20:1_ and C_20:1_ to C_22:1_; (Kunst et al., 1992; Roscoe et al., 2001). Thus, it is possible for the genetic manipulation of *BnFAE* to increase C18 lipid species. Shi et al. (2015) performed RNAi knockdown of *BnFAE*, which resulted in the relative proportion of oleic acid with other fatty acids in seed oil increasing from 21.3% to 60.0% while decreasing the relative proportion of erucic acid from 42.5% to 2.1%. In order to further increase the relative proportion of seed oleic acid, researchers created both *BnFAE1-Ri* and *BnFAD2-Ri* lines. Unlike*BnFAE1-Ri*, the relative proportion of seed oleic acid of *BnFAD2-Ri* increased modestly, from 21.3% to 31.9%, whereas the relative proportion of erucic acid content slightly rose from 42.3% to 45.6%. Interestingly, following hybridization between the two RNAi knockdown mutants, a higher relative proportion of oleic acid was obtained that reached 80.3% in the *BnFAD2-Ri/BnFAE1-Ri* lines (Peng et al., 2010; Shi et al., 2017). In addition to *FAE1*, Zhang et al. (2018) identified a CCCH-type transcription factor (BnZFP1) from a rapeseed subtractive hybridization library that correlated well with seed oleic acid content. Overexpressing *BnZFP1* in rapeseed led to its proportion of seed oleic acid increasing to 65.8% to 75.7%, higher than the wild type’s (63.7%). However, the seed oleic acid content was significantly decreased by 4.8% when the *BnZFP1* expression was interrupted in transgenic plants.

Apart from *BnFAE1* and *BnZFP1*, the *BnFAD2* gene has been studied extensively in oil crops during fatty acid biosynthesis and is directly associated with oleic acid content (Fulvio et al., 2022; Razeghi-Jahromi et al., 2022). We know that disruption of *FAD2* results in a greater oleic acid content of different tissues, such as roots, leaves, and seed in *Arabidopsis* (Okuley et al., 1994). In rapeseed, a major locus responsible for seed oleic acid content on chromosome A5 was found and mapped, it accounting for ca. 83% of the total variation. Furthermore, *BnFAD2* had four copies: *BnaFAD2.A1*, *BnaFAD2.A5*, *BnaFAD2.C1*, and *BnaFAD2.C5* (Yang et al., 2012). Other two loci on chromosomes 3 and 9 were also detected (Zhao et al., 2008; Zhao et al., 2019). Till now, it remains unclear how many minor-effect loci controlling seed oleic acid content exist in rapeseed, but this is not important because of major-effect loci found according to a previous investigation (Yang et al., 2012). Therefore, the selection of a targeted locus is essential when pursuing the high oleic acid content improvement of rapeseed. Sivaraman et al. (2004) developed a high-oleic acid content line in a zero-erucic acid line of mustard (*Brassica juncea* L.) by antisense suppression of the *B. rapa FAD2* gene. The relative proportion of seed oleic acid increased from 39.5%~53.4% to 69.0%~74.8%.

Recently, however, genomic editing technology has gained popularity (Bahariah et al., 2021; Vu et al., 2021). Okuzaki et al. (2018) performed CRISPR-Cas9-mediated genome editing of target gene *BnFAD2_Aa*, which resulted in a higher relative proportion of oleic acid, rising from 74.6% in the wild type to 80.0% in the best-performing line. Later, Huang et al. (2020) developed genome editing with the CRISPR-Cas9 system to simultaneously modify *BnFAD2* multiple copies in order to further increase the seed oleic acid content; it generally reached 75.0%–80.0%, with only two lines having a seed oleic acid content greater than 80.0%. The reason why this multiple modification yielded only two lines with an oleic acid composition of more than 80% is still unknown. In this study, we hypothesized that, compared with editing of a single locus, double editing of the A5 and C5 copies of *BnFAD2 via* the CRISPR/Cas 9 system would significantly increase the seed oleic acid content of rapeseed.

Here, we employed the CRIPR/Cas9 system to mutate A5 and C5 copies of *BnaFAD2* in rapeseed. Successful mutagenesis resulted in higher seed oleic acid content of targeted mutants at *BnaFAD2* vis-à-vis the wild type. Further, the mutated trait can be stably inherited with no significant differences in seed yield and oil content. Our results suggest that precise modification of the double loci of *BnFAD2* can be applied in the CRISPR/Cas9 system to create new germplasms for the purpose of breeding plants with high seed oleic acid content.

## Materials and methods

### Plants

The semi-winter *Brassica napus* line B57-1 was used for *Agrobacterium*-mediated transformation. This line has proven itself highly reliable and effective in the production of transgenic plants. It has high seed oil content (51.23%) and low relative proportion of seed oleic acid (67.25%).

The transformed plants (T_0_) and subsequent plants with different generations (T_1_ and T_4_) for seed fatty acid analysis were grown in a greenhouse. Because there was only one plant of each mutated line in the T_0_ generation, there were no replications in this generation. From T_1_ to T_4_, the mutated plants and wild type (B57-1) were planted in a completely randomized block design with three replications. A mutant line of #289 was selected to compare with the wild type on the yield, yield component, oil content, and relative proportion of oleic acid with other fatty acids in seed oil. Both lines were in a completely randomized block design with three replications as well in greenhouse conditions. The area of the block was 6 m^2^ with a population density of 15,000 plant ha^-1^.

All plants were planted from October 5th to 10th in each generation. Soil was only watered before seeding, and no irrigation was employed after seeding. Plants were thinned into one plant when they were at the three-true leaf stage. Low release compound fertilizer (N-P_2_O_5_-K_2_O: 20-7-8, Hubei Yishizhuang Agricultural Technology Company Ltd., Yichang, China) was applied in a dose of 1,500 kg ha^-1^ as basal fertilizer before seeding. No top dressing was performed. The mean temperature (outside of the greenhouse because we did not record the temperature in the greenhouse) is illustrated in **Figure S1** from 2019 to 2022 during the rapeseed growth season.

### CRISPR/Cas9 target locus selection, construct assembly, and plant transformation

**Figure 1:**
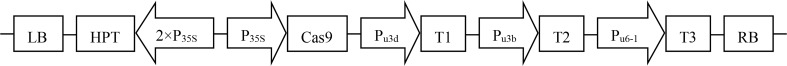
The binary construct *P35S:Cas9-BnFAD2* with three sgRNAs driven by the *U3d*, *U3b*, and *U6-1* promoters from *Arabidopsis*.

Three sgRNAs T1, T2, and T3, targeting both *BnaA05.FAD2* and *BnaC05.FAD2*, were designed online (http://crispr.hzau.edu.cn/CRISPR2). The binary pYLCRIPSR/Cas9 multiplex genome targeting vector system, kindly provided by Prof. Yaoguang Liu (South China Agricultural University), included both pYLCRISPR/Cas9P_ubi_-H and pYLCRISPR/Cas9P_35S_-H and three plasmids with sgRNA cassettes driven by the promoters of *AtU3d*, *AtU3b*, and *AtU6-1*; this system was used for the construct assembly following a previously described methodology (Ma et al., 2015). The oligos used to construct the sgRNA vectors are listed in **Table S1**. The resulting construct contained a Cas9 expression cassette and sgRNA expression cassettes with target sequences and a hygromycin resistance cassette (**Figure 1**).

After verifying the fused constructs by sequencing, the resulting constructs were transformed into *B. napus via* the *Agrobacterium tumefaciens*-mediated hypocotyl method (Zhou et al., 2002). Regenerated seedlings (T_0_ lines) were selected according to their resistance to hygromycin and confirmed by PCR, the latter using the flanking primers PB-L and PB-R (**Table S1**) (Ma et al., 2015).

### Identification of mutated transgenic plants

PCR was performed to amplify the genomic region surrounding the CRISPR target sites, using specific primers (**Table S1**), and the PCR fragments were directly sequenced to identify the mutations. The ensuing sequences were read through sequencing chromatograms and compared with wild-type (WT) sequences to detect the presence of any indels.

### Analysis on the flanking sequences of transformed vector of mutants at T_1_ generation

The T_1_ mutant lines were grown in a greenhouse. Leaves from each individual were collected to extract their DNA for PCR amplifications using the flanking primers PB-L and PB-R of vector (**Table S1**). Those without amplified bands of individuals were selected for further genotyping; the fragments including the indels were amplified and sequenced using specific primers (**Table S1**).

### DNA extraction and sequencing

Total genomic DNAs were extracted from leaf material with a plant genomic DNA kit (Tiangen Biotech, Beijing, China) by following its instructions. The DNA concentration (>50 ng μl^-1^) was measured by a NanoDrop spectrophotometer, and fragmentation was achieved by applying sonication. Then, the fragmented DNA was purified and end-repaired and its sizes determined by gel electrophoresis. Paired-end libraries with insert sizes of 350 bp were prepared following Illumina’s standard genomic DNA library preparation procedure. Next, a control library quality for sequencing was assembled. We sequenced (based on sequencing by synthesis [SBS] technology) the whole genome of rapeseed using the Illumina NovaSeq 6000 platform (Illumina, USA).

### Assembly

Paired-end Illumina raw reads were cleaned by removing adaptors and barcodes and then quality-filtered using the Trimmomatic tool. Reads were trimmed from both ends, and individual bases with a Phred quality score <20 were discarded, as were any reads having more than three consecutive uncalled bases. Entire reads with a median quality score lower than 21 or less than 40 bp in length after trimming were also discarded. After completing the quality filtering process, the reads were mapped to the reference sequence (**Table S2**), using Bowtie2 v.2.2.6. Then, all putative *FAD2* reads mapped to the reference sequence as mentioned above were used for the *de novo* assembly to reconstruct the *FAD2* gene sequence using SPAdes 3.6.1 with iterative K-mer sizes of 55, 87, and 121. *De novo* assembled contigs were concatenated into larger contigs by Sequencher 5.3.2 software (Gene Codes Inc., Ann Arbor, MI, USA), which based on at least a 20-bp overlap and 98% similarity. A “genome walking” technique, using the Unix “grep” function, was implemented to find all remaining reads that could fill any gaps between contigs that did not assemble in the initial set of analyses.

### Multiple-sequence alignment

To carry out the multiple-sequence alignment, DNAMAN sequence analysis software was used. The consensus sequence among all sequences is presented in this study.

### Analysis of sequences of edited regions in the A1 and C1 loci

Selected double-locus-edited mutants and wild types were sequenced for their *BnFAD2* on each A1 and C1copy. These sequences were matched to the *BnFAD2* gene with three edited regions in both A1 and C1 copies.

### Quantitative reverse transcription-polymerase chain reaction analysis

Seeds from five double-locus-edited mutants were collected. The samples were ground into powder with liquid nitrogen. Total RNA was extracted using the RNeasy Plant Mini Kit (QIAGEN, Hilden, Germany) including treatment with DNase I (Takara, Dalian, China). The RNA was used for cDNA synthesis using a two-step RT-PCR kit (Takara, Dalian, China) according to the manufacturer’s instructions. The resultant cDNA was subjected to reverse transcription-polymerase chain reaction (qRT-PCR) analysis (LightCycler^®^ 480 System, Roche, Basel, Switzerland). The primers of *BnFAD2*on A1, C1, A5, and C5 copies and *BnACTIN* (housekeeping gene) are listed in **Table S1**. The transcript amount was calculated according to Livak and Schmittgen’s method (2001).

### Analysis of fatty acid composition of *B. napus* transgenic lines

The WT and different-type homozygous T_0_, T_1_, and T_4_ mutant lines were grown in a greenhouse, each with three biological replicates except for the T_0_ generation. Seeds were harvested to analyze the composition of fatty acids through gas chromatography, as described by Lian et al. (2017).

### Analysis of yield, yield component, and seed oil content of mutant line and wild type

Five plants were randomly selected excluded from border plants for yield component analysis in each block. Yield components including the number of branches and siliques and seed number of per silique were recorded. A 1,000-seed weight was weighed in a balance. Seed yield was recorded in a core area of each block. Seed oil content was analyzed by the near-infrared method.

### Statistic

Statistical analysis of the data was implemented in SPSS software (v17.0, Chicago, IL, USA). Analyses of variance (ANOVAs) of randomized complete block design were performed on the relative proportion of the seed fatty acid profile among edited mutants and wild type in T_1_ and T_4_ generation, respectively; the transcript level relative to *BnACTIN* of four copies of the *BnFAD2* gene on A1, C1, A5, and C5 chromosome; and seed yield, yield components, oil content, and proportion of oleic acid between mutant (#289) and wild types. Mean values of the above experiment were distinguished for significant differences using Duncan’s test at an alpha probability of 0.05.

## Results

### Regeneration and transformation efficiency of line B57-1

**Figure 2:**
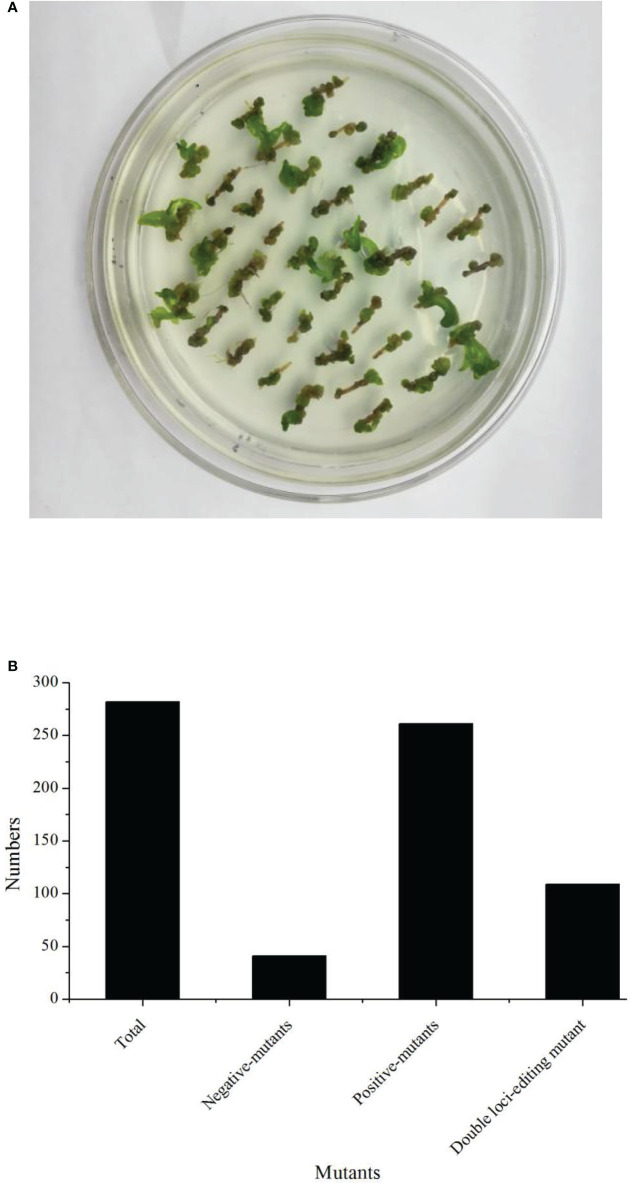
Regeneration status and transformation efficiency of the genotype B57-1. **(A)** Callus from the hypocotyl of B57-1. **(B)** Numbers of plants that underwent total transformation, negative- and positive-mutants.

In this study, we firstly compared the regeneration efficiency of rapeseed with 23 germplasms. These results showed that the regeneration efficiency varied from 0% up to 20.18% (**Table 1**). Eight germplasms had zero regeneration, and three germplasms had a regeneration ratio higher than 10.00%. Therefore, we focused on B57-1, whose hypocotyl served as explant tissue for genetic transformation because of its high regeneration (**Table 1**, **Figure 2A**; **Figure S2**). We further analyzed the number of positive mutants using the CRISPR-Cas9-mediated genome editing of *BnFAD2*. In all, 282 transformants were harvested, consisting of 41 (14.54%) negative mutants and 241 (85.56%) positive mutants (**Figure 2B**). The latter result indicated that the CRIPSR-Cas9-mediated genome editing of *BnFAD2* was successful and highly efficient.

**Table 1:** Regeneration efficiency of rapeseed germplasms.

Germplasms	Explant numbers	Germinated explant numbers after infection	Transformation efficiency of explant %
B6-4	328	5	1.52
B36-5	340	23	6.76
B37-4	334	42	12.57
B47-2	352	0	0.00
B40-2	360	21	5.83
B57-1	337	68	20.18
B69-4	350	28	8.00
B122	320	0	0.00
B123-4	319	0	0.00
B138-3	312	41	13.14
B143-1	315	8	2.54
G196	330	0	0.00
G63-3	352	10	2.84
G75-5	351	32	9.12
G89-2	349	41	11.75
G94-5	322	4	1.24
G93-4	320	6	1.88
G137-1	325	0	0.00
G142-1	335	2	0.60
G145-10	361	0	0.00
G170-3	329	0	0.00
SG119-3	309	0	0.00
SG149-5	326	19	5.83

### Editing type in the CRIPSR-Cas9-mediated genome editing of the *BnFAD2* system

**Figure 3:**
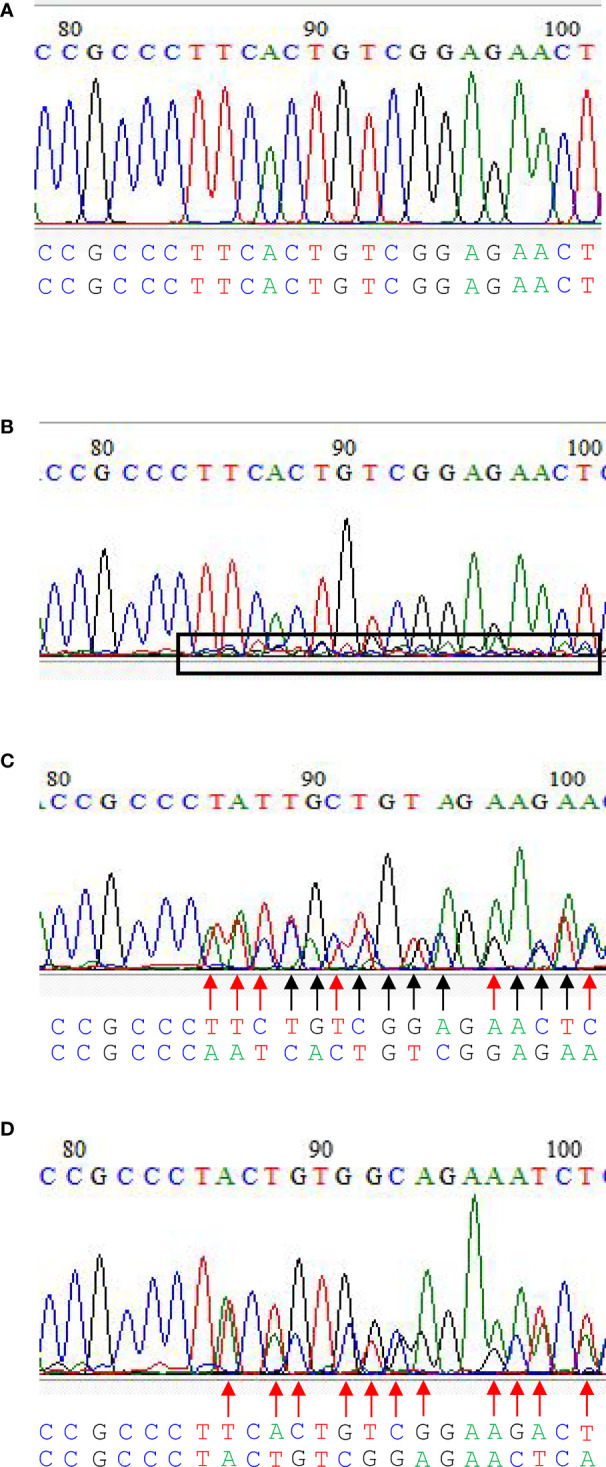
The editing types in CRIPSR/Cas9-mediated genome editing of the double loci of *BnFAD2*. **(A)** Wild type, **(B)** cells partially edited, **(C)** homozygous edited, and **(D)** heterozygous edited. In **(B)**, all the sharp and clear peaks similar to the wild type were observed, which were dominant. Further, some unequal small peaks were observed within the box with a black border. As a callus develops into plant, the phenotype is similar to the wild type, but some mutated tissues exist. Sequences below the figure with the black arrow indicate the base mutated in both homologous chromosomes, while those with the red arrow indicate the base mutated only in one of the homologous chromosomes. Homozygous edited type showed base mutations on both homologous chromosomes compared with the wild type **(C)**. Heterozygous edited type showed base mutations only on one of the homologous chromosome.

Four editing types could be identified in the current CRIPSR-Cas9-mediated genome editing of the *BnFAD2* system. The first type was the wild type, in that *BnFAD2* went unedited by the CRISPR-Cas9-mediated genome editing system (**Figure 3A**). In this type, all peaks of different kinds of base were sharp and clear. The second type was that cells partially edited (**Figure 3B**). In this type, all the sharp and clear peaks the same as the wild type were observed, which was dominant. Further, some unequal small peaks were observed. As a callus develops into plant, the phenotype is similar to the wild type but some mutated tissues exist. Thus, the plant is called chimera. The third type was homozygous, in that the copy was simultaneously edited in a homologous chromosome (**Figure 3C**). In this type, mutated bases can be found in two homologous chromosomes as compared with the wild type (**Figure 3C**). The fourth was heterozygous, whereby a homologous chromosome was mutated but another one was not mutated (**Figure 3D**). In this type, mutated bases can be found in one of the homologous chromosomes. The sequence of another homologous chromosome was the same as the wild type (**Figure 3D**).

### Editing efficiency in the CRIPSR-Cas9-mediated genome editing of the *BnFAD2* system

**Figure 4:**
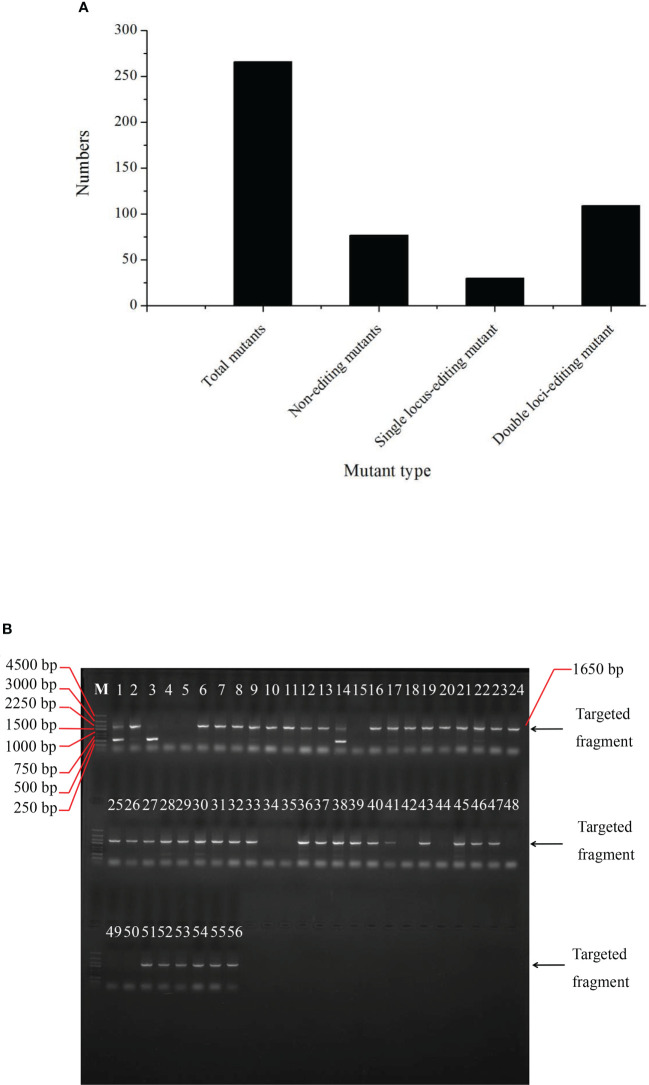
Edited mutant types in the T_0_ generation. **(A)** Numbers of total mutants, non-edited mutant, single locus-edited mutant, and double loci-edited mutant. **(B)** PCR amplification results of T_0_ generation indicating whether the plants numbered from 1 through 56 were edited.

Overall, 216 plants were sequenced to determine the editing efficiency of the CRIPSR-Cas9-mediated genome editing of the *BnFAD2* system. Results showed that 77 lines were not edited by this system (**Figure 4A**). The number of single locus- and double-locus-edited lines was 30 and 109, respectively. Therefore, the total editing efficiency of the CRIPSR-Cas9-mediated genome editing of the *BnFAD2* system was 64.35%, for which the single- and double-locus editing efficiencies were 21.58% and 78.42%, respectively (**Figure 4A**). The PCR results for the edited lines are depicted in **Figure 4B**. Evidently, the edited lines were detected in the targeted band after the electrophoresis.

### Analysis of the fatty acid profile in T_0_ seed of wild-type and genome-edited mutants

We then analyzed the seed fatty acid profile in T_0_ seeds of the wild-type and genome-edited mutants (**Table 2**). The relative proportion of oleic acid with other fatty acids in seed oil was significantly increased in the genome-edited lines whether the line was single locus- or double loci-edited. However, the effect of double-locus-edited mutants on the relative proportion of seed oleic acid was stronger than that of single-locus-edited lines. The relative proportion of seed oleic acid in three single-locus-edited lines, #122, #216, and #294, were below 80%, but it respectively increased by 8.94%, 13.90%, and 15.32%, when compared with the wild type. Furthermore, the two lines #216 and #294 edited on the A5 copy had a similar relative proportion of seed oleic acid that surpassed that of line #122, whose C5 copy had been edited. This suggested that the copy of *BnFAD2* on A5 exerted a greater effect upon seed oleic acid than did the C5 copy. Regarding the double-locus-edited lines, their relative proportion of seed oleic acid spanned 86.99% to 89.61%. The average relative proportion of seed oleic acid of 13 lines was increased by 28.96% vis-à-vis the wild type. As for other fatty acid compounds, the relative proportion of palmitic acid, stearic acid, arachidic acid, and arachidonic acid was each slightly affected by CRIPSR-Cas9-mediated genome editing of the *BnFAD2* system. Furthermore, the erucic acid contents in all mutant lines and wild types were undetectable. The relative proportion of linoleic acid and linolenic acid decreased drastically in the mutants especially for double-locus-edited ones. Averagely, the relative proportions of linoleic acid and linolenic acid were decreased by 81.93% and 63.29%, respectively. Clearly, the bolstered seed oleic acid content arose from the reduction in linolenic acid and linoleic acid contents, albeit differently between single locus- and double loci-edited lines due to the disruption of *BnFAD2*. For the effect of mutation type on seed fatty acid profile in edited mutants, no difference in the proportion of seed oleic acid, linolenic acid, and linoleic acid was observed between a mutant (#202) with a heterozygous type in C5 copy and a wild type. Further, heterozygous type in both-locus (A5/C5)-edited mutants (#106, #112, and #129) showed a decreased proportion of oleic acid, which ranged from 75.97% to 81.86%. The proportion of oleic acid was higher than the wild type but substantially reduced as compared to the homozygous ones (**Table 2**). The result indicated that homozygous mutants contributed more to the percent increase in seed oleic acid compared to the wild type.

**Table 2:** Fatty acid profile in T_0_ seed of wild type (WT, B57-1) and genome-edited mutants (%).

Mutant	Locus	Mutation type	C_16:0_	C_18:0_	C_18:1_	C_18:2_	C_18:3_	C_20:0_	C_20:1_
B57-1	WT	–	4.43	0.96	68.33	18.71	6.18	0.36	1.03
#19	A5/C5	Homozygous	3.67	0.79	87.63	4.09	2.10	0.40	1.32
#105	A5/C5	Homozygous	3.60	1.05	87.91	3.47	2.42	0.31	1.24
#106	A5/C5	Heterozygous	4.56	1.07	75.97	10.01	7.27	0.30	0.82
#112	A5/C5	Heterozygous	4.28	0.96	81.86	6.42	5.24	0.30	0.94
#122	C5	Homozygous	4.06	0.83	74.44	12.85	6.26	0.33	1.23
#129	A5/C5	Heterozygous	4.27	1.02	78.48	8.94	6.00	0.29	1.00
#202	C5	Heterozygous	4.14	0.87	68.86	18.24	6.20	0.40	1.29
#206	A5/C5	Homozygous	3.84	0.67	86.99	3.91	2.91	0.32	1.36
#215	A5/C5	Homozygous	3.43	0.60	88.55	3.58	2.04	0.31	1.49
#216	A5	Homozygous	4.60	0.76	77.83	9.80	5.38	0.39	1.24
#222	A5/C5	Homozygous	3.39	0.56	88.24	3.73	2.20	0.31	1.57
#230	A5/C5	Homozygous	3.21	0.76	88.82	3.47	1.86	0.34	1.54
#238	A5/C5	Homozygous	3.45	0.58	86.43	4.05	3.58	0.31	1.60
#240	A5/C5	Homozygous	3.58	0.81	88.25	3.73	1.76	0.40	1.47
#265	A5/C5	Homozygous	3.18	0.67	89.61	3.11	1.80	0.30	1.33
#273	A5/C5	Homozygous	3.54	0.83	87.96	3.46	2.28	0.38	1.55
#289	A5/C5	Homozygous	3.33	0.58	88.45	3.64	2.15	0.29	1.56
#294	A5	Homozygous	4.16	0.77	78.80	9.74	4.80	0.47	1.26
#314	A5/C5	Homozygous	3.36	0.75	88.38	3.52	2.08	0.39	1.52
#342	A5/C5	Homozygous	3.21	0.59	88.32	3.69	2.32	0.30	1.57

C_22:1_ was not detected in the seed samples of WT as well as mutants.

### Analysis of the seed fatty acid profile in T_0_, T_1_, and T_4_ generation of wild-type and genome-edited mutants

**Figure 5:**
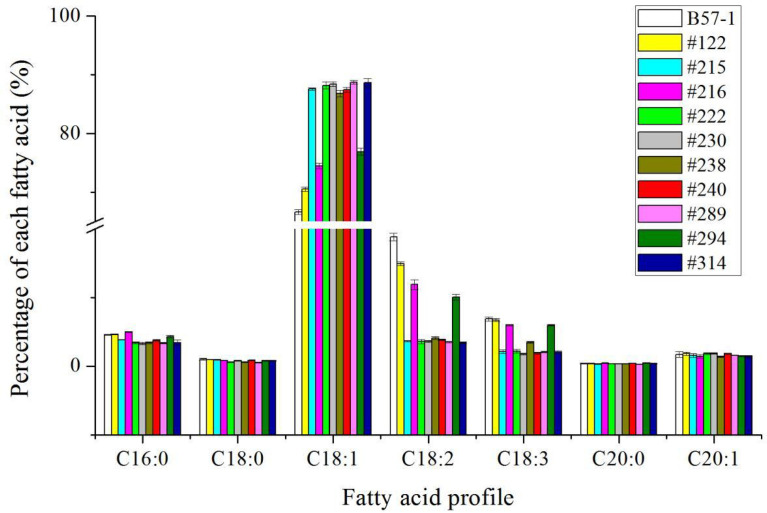
Fatty acid profile of mutants and wild type in T_1_ generation.

**Figure 6:**
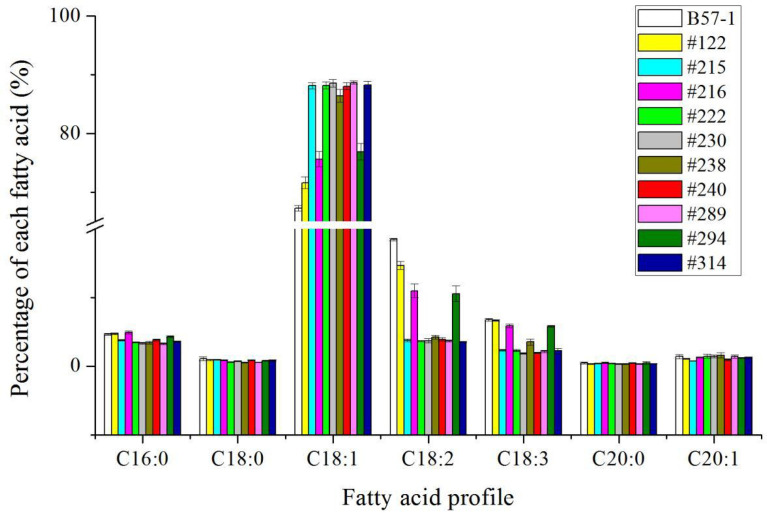
Fatty acid profile of mutants and wild type in T_4_ generation.

To further confirm whether the mutants can be stably inherited from the generations of T_0_, the relative proportion of seed oleic acid was measured in the T_1_ and T_4_ generations (**Figures 5** and **6**; **Table S3** and **S4**). According to these results, the relative proportion of seed oleic acid of both the single-locus- and double-locus-edited lines showed that this trait could be stably inherited. Yet, the relative proportion of seed oleic acid of single locus-edited lines decreased more in the T_1_ generation, by 5.27%, 4.29%, and 2.44% in lines #122, #215, and #294, respectively, but only slightly from the T_1_ to T_4_ generation in those lines. In stark contrast to single-locus-edited lines, double-locus-edited lines exhibited a very stable level of the relative proportion of seed oleic acid with evidence of little variation. The results suggested that the double-locus mutation was more advantageous to the genetic stability of seed oleic acid content than was single locus editing in this study.

### Mutation locus analysis on the edited lines

To further understand the types of editing that give rise to the mutants, five double-edited mutants were selected and their sequences of *BnaFAD2* copies on the A5 and C5 chromosomes were assayed (**Table 3**). Three editing types of the *BnaFAD2* copy on chromosome A5 were distinguishable. The first one entailed a deletion of bases with different base numbers, such as for lines #206 and #342. The second type was insertion of bases such as line #238, at which a base, T, was inserted into one of the homologous chromosomes. The third type was a hybrid, featuring both insertion and deletion of bases such as in lines #230 and #240. The #230 line had the same editing mode whereas different editing modes were discernible in the #240 line, consisting of deletion and insertion/deletion.

**Table 3:** Mutation types of selected mutants on the A5 and C5 copies of the *BnFAD*2 gene.

Mutant	A5 copy of *FAD2*	Mutation type	C5 copy of *FAD2*	Mutation type
	Original sequence: CCGCCCTTCACTGTCGGAGAACT			
206	CCGCCCTT-ACTGTCGGAGAACT	-1	CCGCCC-TCACTGTCGGAGAACT	-1
	CCGCCC––C-C-GTCGGAGAACT	-4	CCGCC-TT––CTGTCGGAGAACT	-3
230	CCGCCC**C**–––––TGTCGGAGAACT	+1/-5	CCGCCCTT––––GTCGGAGAACT	-4
	CCGCCC**C**–––––TGTCGGAGAACT	+1/-5	CC––––**A**-TCAC-GTCGGAGAACT	+1/-6
238	CCGCCCTT**T**CACTGTCGGAGAACT	+1	CCGCCC––––––GTCGGAGAACT	-6
	CCGCCC–––A––––CGGAGAACT	-7	CCGCCC-TC––––GTCGGAGAACT	-4
240	CCGCCCTTC–––GTCGGAGAACT	-3	CCGCCCT––––––––––––––//	-348
	CCGCCC**G**––––CT–––GGAGAACT	+1/-7	CCGCCCT––––––––––––––//	-348
342	CCGCCC––––––GTCGGAGAACT	-6	CCGCCCT**AT-//-AT**TCACTGTCGGAGAACT	+400
	CCGCC––TC––TGTCGGAGAACT	-4	CCGCCCT**AT-//-AT**TCACTGTCGGAGAACT	+400

The uppercase with bold indicates the base(s) was inserted in the edited mutants.

Four editing types of the *BnaFAD2* copy on chromosome C5 were found. The first was deletion of bases with different base numbers, such as for lines #206 and #238. The second type was the hybrid (both insertion and deletion of bases) such as the #230 line. The third was large-fragment deletion, such as in the case of line #240 for which a fragment having 348 bases was deleted. The fourth type was large-fragment insertion, for example, as happened in the #342 line, with the insertion of a fragment comprising 400 bases. These results suggested that not solely one type but rather multiple editing modes can generate a seed high oleic acid content.

### Analysis on the flanking sequences of transformed vector of mutants from the T_1_ generation

To confirm whether the mutants from T_1_ generation contained the fragment of the transformed vector, five mutants were selected (#407, #397, #486, #510, and #382) for re-sequencing analysis. Results showed that although sequencing reads can be matched to the sequence of the targeted gene, most could only match to ca. the 1,287th position therein. Furthermore, at this position, only 30 bp can be reliably matched. Hence, it may be concluded that no reads can be matched to the vector sequence (**Table 4**). The finding suggested that the segregation of mutants happened without the fragment from the transformed vector.

**Table 4:** Transgenic analysis on the mutants *via* matching between sequenced reads and the reference sequence at the 1,287th position.

ID of sequenced reads	CIGAR^a^	Sequence of reads matched to reference sequence
A001596:661:HCHL2DSXY:3:1101:10420:18912	41S30M79S^b^	ATGACAGGGGAGCCGGCGACCGAAGCCCCGGTGAACGGCGGCCGTAACTATAAC GGTCCTAAGGTAGCGAAATTCCTTGTCGGGTAAGTTCCGACCCGCACGAAAGGC
A001596:661:HCHL2DSXY:3:1101:24243:24533	10S30M110S	TGAACGGCGGCCGTAACTATAACGGTCCTAAGGTAGCGAAATTCCTTGTCGGGT AAGTTCCGACCCGCACGAAAGGCGTAACGATCTGGGCACTGTCTCGGAGAGAGG
A001596:661:HCHL2DSXY:3:1101:7166:25520	80S30M40S	CCTGCCCAGTGCCGGTAGGTCAAGGTAGTTGGTGACCTGATGACTGGGGAGCCG GCGACCGAAGCCCCGGTGAACGGCGGCCGTAACTATAACGGTCCTAAGGTAGCG
A001596:661:HCHL2DSXY:3:1101:26811:32456	55S30M65S	AAGTTGGTGACCTGATGACAGGGGAGCCGGCGACCGAAGCCCCGGTGAACGGCG GCCGTAACTATAACGGTCCTAAGGTAGCGAAATTCCTTGTCGGGTAAGTTCCGA
A001596:661:HCHL2DSXY:3:1101:23484:33426	31S30M89S	AGCCGGCGACCGAAGCCCCGGTGAACGGCGGCCGTAACTATAACGGTCCTAAGG TAGCGAAATTCCTTGTCGGGTAAGTTCCGACCCGCACGAAAGGCGTAACGATCT
A001596:661:HCHL2DSXY:3:1101:20428:36057	62S30M58S	GTCAAGGAAGTTGGTGACCTGATGACAGGGGAGCCGGCGACCGAAGCCCCGGTG AACGGCGGCCGTAACTATAACGGTCCTAAGGTAGCGAAATTCCTTGTCGGGTAA
A001596:661:HCHL2DSXY:3:1101:8802:36993	65S30M55S	AAGGTCAAGGAAGTTGGTGACCTGATGACAGGGGAGCCGGCGACCGAAGCCCCG GTGAACGGCGGCCGTAACTATAACGGTCCTAAGGTAGCGAAATTCCTTGTCGGG
A001596:661:HCHL2DSXY:3:1101:9218:4679	65S30M55S	AAGGTCAAGGAAGTTGGTGACCTGATGACAGGGGAGCCGGCGACCGAAGCCCCG GTGAACGGCGGCCGTAACTATAACGGTCCTAAGGTAGCGAAATTCCTTGTCGGG
A001596:661:HCHL2DSXY:3:1101:30472:10645	17S30M103S	GCCCCGGTGAACGGCGGCCGTAACTATAACGGTCCTAAGGTAGCGAAATTCCTT GTCGGGTAAGTTCCGACCCGCACGAAAGGCGTAACGATCTGGGCACTGTCTCGG
A001596:661:HCHL2DSXY:3:1101:31015:16282	17S30M103S	GCCCCGGTGAACGGCGGCCGTAACTATAACGGTCCTAAGGTAGCGAAATTCCTT GTCGGGTAAGTTCCGACCCGCACGAAAGGCGTAACGATCTGGGCACTGTCTCGG

^a^Abbreviation for the Compact Idiosyncratic Gapped Alignment Report. ^b^S means for base shearing, M means the base was matched. For example, 41S30M79S means 41 bases were sheared and removed, 30 bases were matched to the reference sequence, and the last 79 bases were sheared and removed.

### Sequence analysis of *BnFAD2* on A1 and C1 copies

Because there were four copies of *BnFAD2* identified in previous research (Huang et al., 2020), their sequences in the selected lines were further compared to confirm whether the copies of *BnFAD2* on A1 and C1 were indeed edited. Results showed that the three editing targeted regions lacked any sequence changes, suggesting no editing occurred on A1 (**Table 5**; **Figure S3**). Concerning the copy on the C1 locus, the two editing targeted regions from positions 236 to 258 and from positions 728 to 750 both had one base in each line that differed from the sequence of *BnFAD2* (**Table 6**; **Figure S4**). However, this differing base in all edited lines was the same as that in the wild type. Therefore, we may tentatively presume no editing occurred in either region. For another region, this spanning the positions 575 to 596, a deletion/insertion occurred in two places, at the 575th and 592nd positions. At the 575th position, three mutant lines incurred a deletion of “C”, the same as for the wild type. Hence, this position was difficult to judge whether it was an editing position. However, at the 592nd position, in two lines there was an “A” insertion. This position was regarded as edited position. At the 596th position, two lines showed the base substitution of “C” to “T” and this position could be regarded as edited position.

**Table 5:** Sequence comparison among references of *BnFAD2*, wild type, and mutants in the targeted editing region of the A1 copy.

Mutant	Genome editing targeted region1	Genome editing targeted region 2	Genome editing targeted region 3
*FAD2*	CCACCATTCACTCTCGGAGACCT	TGACGCCACCATTCCAACACCGG	TACTTAGCCTTCAACGTCTCGGG
H3cp3-2	CCACCATTCACTCTCGGAGACCT	TGACGCCACCATTCCAACACCGG	TACTTAGCCTTCAACGTCTCGGG
H3cp4-2	CCACCATTCACTCTCGGAGACCT	TGACGCCACCATTCCAACACCGG	TACTTAGCCTTCAACGTCTCGGG
H3cp5-2	CCACCATTCACTCTCGGAGACCT	TGACGCCACCATTCCAACACCGG	TACTTAGCCTTCAACGTCTCGGG
H3cp6-2	CCACCATTCACTCTCGGAGACCT	TGACGCCACCATTCCAACACCGG	TACTTAGCCTTCAACGTCTCGGG
H3cp7-2	CCACCATTCACTCTCGGAGACCT	TGACGCCACCATTCCAACACCGG	TACTTAGCCTTCAACGTCTCGGG
H3cp8-2	CCACCATTCACTCTCGGAGACCT	TGACGCCACCATTCCAACACCGG	TACTTAGCCTTCAACGTCTCGGG
Consensus	CCACCATTCACTCTCGGAGACCT	TGACGCCACCATTCCAACACCGG	TACTTAGCCTTCAACGTCTCGGG

**Table 6:** Sequence comparison among references of *BnFAD2*, wild type, and mutants in the targeted editing region of the C1 copy.

Mutant	Genome editing targeted region 1	Genome editing targeted region 2	Genome editing targeted region 3
*FAD2*	CCACCCTTCACTCTCGGAGACCT	CGACGCCACCATTCCAA.CACC	TACTTAGCCTTCAACGTCTCTAA
H3cp3-2	CCACCATTCACTCTCGGAGACCT	.GACGCCACCATTCCAA.CACC	TACTTAGCCTTCAACGTCTCGAA
H3cp4-2	CCACCATTCACTCTCGGAGACCT	.GACGCCACCATTCCAA.CACC	TACTTAGCCTTCAACGTCTCGAA
H3cp5-2	CCACCATTCACTCTCGGAGACCT	CGACGCCACCATTCCAA.CACT	TACTTAGCCTTCAACGTCTCGAA
H3cp6-2	CCACCATTCACTCTCGGAGACCT	.GACGCCACCATTCCAA.CACC	TACTTAGCCTTCAACGTCTCGAA
H3cp7-2	CCACCATTCACTCTCGGAGACCT	CGACGCCACCATTCCAAACACT	TACTTAGCCTTCAACGTCTCGAA
H3cp8-2	CCACCATTCACTCTCGGAGACCT	.GACGCCACCATTCCAAACACC	TACTTAGCCTTCAACGTCTCGAA
Consensus	CCACC-TTCACTCTCGGAGACCT	-GACGCCACCATTCCAA-CAC-	TACTTAGCCTTCAACGTCTC-AA

### Analysis on the transcript of *BnFAD2* in seed of the mutants by qRT-PCR

**Figure 7:**
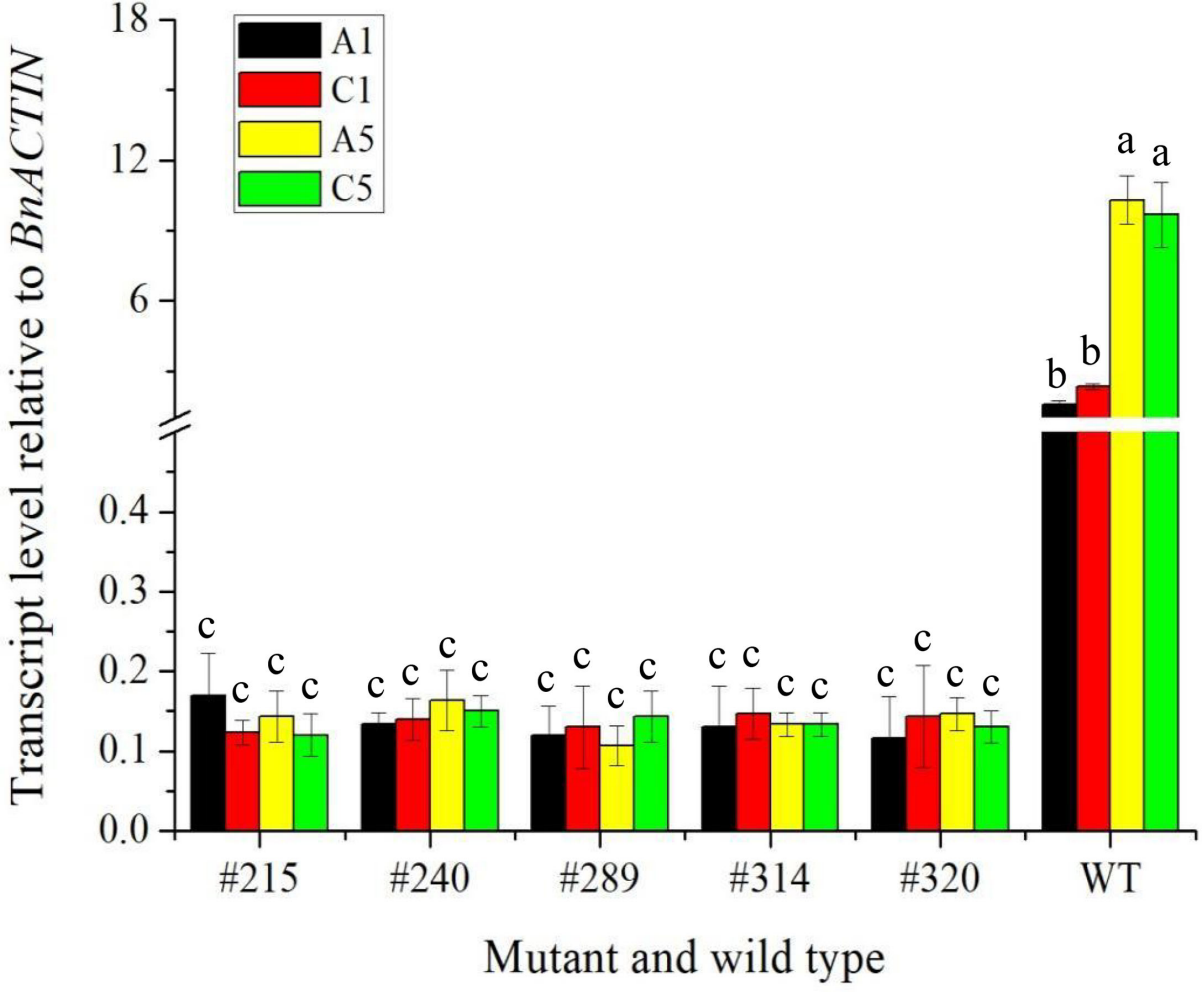
The transcript level relative to *BnACTIN* of four copies of the *BnFAD2* gene on A1, C1, A5, and C5 chromosome in the five edited mutants and wild type *via* qPCR. The different lowercase above the histogram indicates a significant difference at the 0.05 probability level.

To confirm whether the expression of the edited gene, *BnFAD2*, was affected in A1, C1, A5, and C5 copies in seeds of five selected double-edited mutants using wild type as a control, a qRT-PCR analysis was performed (**Figure 7**). Results showed that the difference in the transcript level of *BnFAD2* in the A5 and C5 copies was not significant in the wild type. The same trend was found in the A1 and C1 copies. However, the transcript levels of *BnFAD2* in both A1 and C1 copies were significantly lower than those in the A5 and C5 copies in the wild type. Interestingly, the transcript levels of *BnFAD2* of four copies in all mutants were significantly reduced as compared with the wild type. The results suggested that the expression of the *BnFAD2* gene was markedly inhibited after the gene was edited.

### Yield, yield components, and seed oil content analysis between wild-type and edited line

To further understand whether genome editing affected the seed yield, oil content, or oleic acid content, a line (#320) (**Figure S5**) was selected to compare those traits with the wild type (**Table 7**). No significant differences for the seed yield component, including plant branch numbers, seed numbers per silique, and 1000-seed weight, were found. Compared with the wild type, the number of siliques per plant was significantly lower in the edited line (reduced by 3.11%). Seed yield and seed oil content were similar between the edited line and the wild type.

**Table 7:** Seed yield, yield components, seed oil content, and proportion of oleic acid between wild type (B57-1) and a selected mutant (#289).

lines	Branches numbers per plant	Siliques per plant	Seed numbers per silique	1000-seed weight (g)	Seed yield kg ha^-1^	Oil content (%)	Oleic acid content (%)
B57-1	8.3a	379.4a	21.42a	3.89a	3465.2a	51.23a	67.25b
#289	8.0a	367.6b	21.51a	3.95a	3429.3a	51.19a	88.56a

Lowercase letters next to the numbers in one column represent significant differences, p ≤ 0.05.

## Discussion

Although there are many ways by which one or more traits were enhanced in crops to increase crop yield and quality, the efficiency among these methods can be drastically different (Saker and Singh, 2015; Khosa et al., 2016). Mutations introduced *via* physical, chemical, or biotechnological methods are useful tools in crop breeding (Mahadtanapuk et al., 2014; Jung and Till, 2021; Tamilzharasi et al., 2022). In order to increase seed oleic acid content, previous investigations indicate two methods that have been extensively applied, which are chemical mutation (EMS) and biological mutation (Auld et al., 1992; Sivaraman et al., 2004; Peng et al., 2010; Lee et al., 2018). Physical mutation from irradiation using ^60^Co and γ rays was not reported to improve the seed oleic acid content in rapeseed. This may be due to the low possibility of a desirable mutation ensuing because the mutation mechanism is a fragment disruption of the chromosome that results in a random form (Gaulden and Read, 1978). Furthermore, the disrupted chromosome fragment carrying the target gene will correspondingly discard many closely linked genes, which could affect other traits positively or negatively. Unlike physical mutation in crops, chemical mutation has been applied more frequently to rapeseed, including its seed oleic acid content. Over the decades, many studies have demonstrated that EMS mutagenesis provides an effective way to improve seed oleic acid content as well as other traits (Lee et al., 2018; Patel et al., 2022). Furthermore, many researchers were able to obtain plants whose seed oleic acid content was more than 85% (Auld et al., 1992). Nevertheless, EMS mutagenesis also occurs in a random form, and to be successful, it requires a large screening population, which is laborious work.

Accordingly, the application of biotechnological mutation is becoming increasingly attractive because of its high efficiency and accuracy and comparatively less labor involved. It is clear that RNAi can increase seed oleic acid content by reducing the erucic acid content *via* manipulation of *BnFAE* (Shi et al., 2015); however, the content of seed oleic acid increased to no more than ca. 65%, yet it could not go higher despite the manipulation of *BnFAE*. We know that after low erucic acid resource is introduced into other germplasms, all of the released rapeseed varieties should feature a low erucic acid content, and currently released rapeseed varieties do have a seed oleic acid content about 65%. Therefore, as is, there is nearly no room to improve seed oleic acid content by further manipulation of *BnFAE*. Another phenomenon should be highlighted: when manipulating *BnFAD2* against a high erucic acid background, although the seed oleic acid content significantly increased, the seed erucic acid increased slightly in comparison with the wild type (Shi et al., 2017). The phenomenon is interesting in that when *BnFAE* was knocked down in plants, the contribution of increased oleic acid content was from the reduction in erucic acid content. However, when *BnFAD2* was knocked down in plants with high erucic acid content, the contribution of increased oleic acid content was from the reduction in linoleic acid and linolenic acid contents (Shi et al., 2017). Thus, the mechanism of the manipulation on *BnFAE* and *BnFAD2* should be different.

In addition to RNAi, genome editing by the CRISPR/Cas 9 system is another powerful biotechnological system for crop trait improvement. In the CRISPR/Cas9 system, selection of appropriate genotypes with high efficiency of regeneration is essential. In rapeseed, Westar is a robust genotype for transformation and it is widely used in research, such as gene function analysis (Kang et al., 2021; Hong et al., 2022). However, because Westar is not a variety from China, obtaining a desirable yield and quality is often not possible due to suboptimal growing conditions for it in China. Therefore, we screened new germplasms to find one capable for high efficiency of transformation, this being a semi-winter type of rapeseed. Apart from the screened germplasm in our study, other researchers have also used their own crop genotype instead of Westar (Shi et al., 2015; Huang et al., 2020). Because of the high efficiency of transformation, the CRISPR/Cas9 system was successfully established with double-locus editing. For editing efficiency in our study, it had a high percentage of 64.35% and the main type was double-locus-edited mutants (**Figure 4A**). It was reported that editing efficiency varied drastically from 5.3% to 100% per transformant with different vectors to edit three genes in rapeseed (Yang et al., 2017). For another example, the editing efficiency of *pFAD2_Aa1* and *pFAD2_Aa2* vectors in rapeseed was 5% and 50%, respectively (Okuzaki et al., 2018). In addition to selection of vector, the targeting efficiency of sgRNA was also affected by other factors such as target sequence and secondary structure of the targeted sgRNA (Ma et al., 2015; Scheben et al., 2017). In our result, the higher editing efficiency is possibly relevant to the high specificity of the sgRNA sequence, which can avoid off-target events greatly. Secondly, the selected editing site is suitable for nucleotide cutting with no disturbance by other proteins and Cas9 can easily approach to the site. However, the speculation required further experimental evidence. Therefore, selection of an appropriate vector, specific sgRNA sequence, and editing sites might be crucial to boost editing efficiency in rapeseed. For the edited mutation type, four types were observed in our study, which was in accordance with a previous study (Nakazato et al., 2021). It was reported that complex editing patterns were found including complete, partially, and entirely absent editing in seedlings of *Arabidopsis*. However, the ratio of chimeric mutation was low, and the percentage of homozygous mutants reached 86.4% in T_1_ plants (Nakazato et al., 2021). Thus, it is reasonable to emphasize on the homozygous mutants in the study.

Although Okuzaki et al. (2018) reported that seed oleic acid content can be increased to 80.0% by editing of *BnFAD2* in the CRISPR/Cas9 system, it is unclear how many or which copies were actually edited. It is quite possible it was the A5 copy because the reported seed oleic acid content was 79.0%~80.0% which concurs with our results (**Table 3**, **Figures 5** and **6**) and previous investigation (Huang et al., 2020). In the present results, the A5 locus of *BnFAD2* elicited a greater effect than did its C5 locus, which was consistent with previous results (Huang et al., 2020). Although our goal was to edit two loci on A5 and C5, the double-locus editing efficiency was high, reaching 78.4% in the mutants. Furthermore, the seed oleic acid content of all selected mutants with double loci exceeded 85%. In addition to the A5 and C5 loci, researchers have identified two other copies on A1 and C1 (Yang et al., 2012; Huang et al., 2020). Our results show that no editing issues arose for A1 in this study. As for the C1 copy, only one position showed an insertion with two mutant lines, leaving other regions and positions unedited. These results suggest it is credible to use the precise design of the double-locus editing system. Regardless, it was suggested that seed oleic acid content can be higher than that attained by single-locus editing (Huang et al., 2020); however, they only got two lines with the relative proportion of seed oleic acid >80% with both loci edited on A5 and C5. The reasons for this discrepancy are unclear because the mutation type was unknown for double edited loci in the previous study. In our study, many types of mutation were found. Multiple mutation forms were also found in other investigations (Okuzaki et al., 2018; Huang et al., 2020; Trinh et al., 2022). The results suggest that the mutation type may not be a decisive factor for determining the augmented seed oleic acid content. Although genome editing with the CRISPR/Cas9 system is an efficient method in crop trait improvement, the system has a risk of off-target resulting undesirable mutations because of the similar sequences between on-target and off-target sites (Park et al., 2022; Wu et al., 2022). Many strategies were attempted to reduce genome-wide off-target events and made great achievements (Fu et al., 2014; Kleinstiver et al., 2016; Zhang et al., 2019). As a result, considerable reports showed that off-target events were not detected in the CRISPR/Cas9 system including improvement of seed oleic acid content in rapeseed (Jia et al., 2017; Okuzaki et al., 2018; Wang et al., 2018; Zhang et al., 2019; Huang et al., 2020). Non-detection of off-target events in the previous study of genome editing of *BnFAD2* might be due to a reliable CISPR/Cas9 system and small numbers of *BnFAD2* copy. Therefore, we did not perform off-target analysis in the current study because of its low possibility.

Because the usage of the CRISPR/Cas9 system is designed to target one trait, it is not a desirable outcome if other important crop traits such as yield and quality are reduced. Therefore, we also compared the seed yield and yield component between the wild type and mutant with high seed oleic acid content. We found no significant differences in terms of seed yield and oil content. However, the number of siliques decreased significantly in the mutant. The yield of rapeseed is composed of the number of siliques, seed number per silique, and seed weight. Here, the markedly decreased number of siliques did not lead to the significantly decreased seed yield in the mutant. One of the possible reasons was that yield components including the number of siliques, seed number per silique, and seed weight analysis were on a single-plant-basis method. Those siliques in a plant usually contain different amounts of seed. Further, seed number per silique was not measured from all of the siliques. Therefore, the reliability of yield components may be lower in our traditional analysis. Differently from yield component analysis, seed yield analysis was based on the population level, with the seeds from all plants and all siliques in a block. Therefore, it should be more reliable for the result of seed yield. For the proportion of seed oleic acid content, the edited mutant was significantly higher than that in the wild type. According to fatty acids changed in the edited mutants, it can be easily found that the contribution of a higher oleic acid proportion was mainly due to the reduction in linolic acid content in one-locus-edited mutants. However, both reduction in linolenic acid and linolic acid content in double-locus-edited mutants led to a very high proportion of oleic acid. Fatty acid desaturase 2 is an important enzyme that catalyzes oleic acid converting into linolenic acid (Lee et al., 2013). Results of the gene expression analysis of *BnFAD2* showed that the transcript level was very low when *BnFAD2* was knocked out in the edited mutants. Thus, the conversion of oleic acid into linolenic acid was blocked resulting in the higher oleic acid content in single-locus-edited mutants. However, in the double-locus-edited mutants, the linoleic acid content was also decreased. It might be due to the influence on the expression of *FAD3*, which is responsible for the conversion from linolenic acid to linoleic acid (Wallis and Browse, 2002). However, further experiment is needed to clarify this inference. These results indicated that the derived mutant *via* editing can be directly used for further production in additional breeding programs because of the similar seed yield trait to the wild type.

In conclusion, we screened a genotype with high regeneration and transformation efficiency that we used for establishing the CRISPR/Cas9-mediating genome editing of double loci of *BnFAD2*. The stronger effect of the A5 locus than C5 locus upon seed oleic acid content was demonstrated, and further improvement of seed oleic acid content was successful by editing both loci. The mutants underwent precise editing of their A5 and C5 copies. Importantly, whereas the resulting mutant had higher seed oleic acid content, its seed yield and oil content were not affected by genome editing.

## Data availability statement

The original contributions presented in the study are publicly available. This data can be found here: NCBI, PRJNA879079.

## Author contributions

SH designed the experiment and revised the experiment. HL performed the experiment (all) and wrote the manuscript. BL, PH, LH, and BX performed the transformation experiment and PCR detection of mutants. YR performed the fatty acid analysis and yield and oil content comparison between wild types and mutants. LJ and YZ assisted on the CRISPR/Cas9 system establishment. All authors approved the final version of the article.

## Funding

This work was supported by the National Natural Science Foundation of China (32130076), the earmarked fund for the China Agriculture Research System (CARS-12), and the Zhejiang Key Laboratory of Digital Dry Land Crops (2022E10012).

## Acknowledgments

Great appreciation is given to the editor and reviewers’ critical comments on the improvement of the manuscript.

## Conflict of interest

The authors declare that the research was conducted in the absence of any commercial or financial relationships that could be construed as a potential conflict of interest.

## Publisher’s note

All claims expressed in this article are solely those of the authors and do not necessarily represent those of their affiliated organizations, or those of the publisher, the editors and the reviewers. Any product that may be evaluated in this article, or claim that may be made by its manufacturer, is not guaranteed or endorsed by the publisher.
