# Supplementary material for: CRISPR/Cas9-mediated editing of double loci of BnFAD2 increased the seed oleic acid content of rapeseed (Brassica napus L.)
Source: Front Plant Sci. 2022 Nov 22;13:1034215. doi: 10.3389/fpls.2022.1034215 (PMC9723152; doi:10.3389/fpls.2022.1034215)
Supplement: Supplementary file 1 [file DataSheet_1.docx]

Table S3 Fatty acid profile of T_0_, and T_1_ seed of wild type (WT, B57-1) and genome edited mutants

| Mutant | Locus | T_0_  (C_18:1_) | T_1_ generation | | | | | | | |
| --- | --- | --- | --- | --- | --- | --- | --- | --- | --- | --- |
|  |  |  | C_16:0_ | C_18:0_ | C_18:1_ | C_18:2_ | C_18:3_ | C_20:0_ | C_20:1_ | C_22:1_ |
| B57-1 | CK | 68.33 | 4.56±0.09ab | 1.01±0.12a | 66.62±0.38d | 18.83±0.55a | 6.87±0.30a | 0.42±0.07abc | 1.69±0.38a | - |
| #122 | C5 | 74.44 | 4.67±0.07ab | 0.97±0.06ab | 70.52±0.39c | 14.93±0.30b | 6.73±0.26a | 0.37±0.03bc | 1.81±0.23a | - |
| #215 | A5/C5 | 88.55 | 3.85±0.05c | 0.92±0.06abc | 87.57±2.19a | 3.66±0.10e | 2.12±0.27d | 0.32±0.04c | 1.56±0.30a | - |
| #216 | A5 | 77.83 | 4.98±0.04a | 0.83±0.05abc | 74.49±1.47b | 11.88±0.74c | 5.98±0.10b | 0.44±0.04ab | 1.40±0.26a | - |
| #222 | A5/C5 | 88.24 | 3.38±0.13de | 0.57±0.07d | 88.16±0.61a | 3.58±0.29e | 2.15±0.27d | 0.33±0.03bc | 1.83±0.16a | - |
| #230 | A5/C5 | 88.82 | 3.29±0.15e | 0.79±0.05bc | 88.37±0.40a | 3.61±0.15e | 1.77±0.12d | 0.32±0.03c | 1.85±0.11a | - |
| #238 | A5/C5 | 86.43 | 3.39±0.12de | 0.57±0.06d | 86.76±1.56a | 4.11±0.21e | 3.47±0.14c | 0.32±0.01c | 1.38±0.11a | - |
| #240 | A5/C5 | 88.25 | 3.76±0.11cd | 0.85±0.04abc | 87.46±1.40a | 3.84±0.09e | 1.88±0.13d | 0.41±0.04abc | 1.80±0.11a | - |
| #289 | A5/C5 | 88.45 | 3.36±0.11de | 0.52±0.04d | 88.70±0.31a | 3.51±0.12e | 2.06±0.11d | 0.27±0.02c | 1.58±0.05a | - |
| #294 | A5 | 78.80 | 4.33±0.14b | 0.81±0.05bc | 76.88±1.61b | 10.06±0.42d | 5.97±0.13b | 0.49±0.07a | 1.46±0.12a | - |
| #314 | A5/C5 | 88.38 | 3.42±0.37cde | 0.77±0.05c | 88.62±0.69a | 3.41±0.12e | 2.01±0.21d | 0.38±0.04abc | 1.39±0.14a | - |

Table S4 Fatty acid profile of T_0_, and T_4_ seed of wild type (WT, B57-1) and genome edited mutants

| Mutant | Locus | T_0_  (C_18:1_) | T_4_ generation | | | | | | | |
| --- | --- | --- | --- | --- | --- | --- | --- | --- | --- | --- |
|  |  |  | C_16:0_ | C_18:0_ | C_18:1_ | C_18:2_ | C_18:3_ | C_20:0_ | C_20:1_ | C_22:1_ |
| B57-1 | CK | 68.33 | 4.61±0.14ab | 1.09±0.26a | 67.26±0.46d | 18.45±0.20a | 6.74±0.20a | 0.45±0.12a | 1.40±0.31ab | - |
| #122 | C5 | 74.44 | 4.73±0.14a | 0.93±0.10ab | 71.62±0.99c | 14.68±0.59b | 6.64±0.10a | 0.33±0.09a | 1.07±0.08abc | - |
| #215 | A5/C5 | 88.55 | 3.76±0.11cd | 0.95±0.09ab | 88.12±0.54a | 3.74±0.21d | 2.32±0.18d | 0.37±0.07a | 0.74±0.03c | - |
| #216 | A5 | 77.83 | 4.91±0.21a | 0.88±0.06ab | 75.61±1.32b | 10.97±1.00c | 5.86±0.29b | 0.48±0.10a | 1.29±0.08abc | - |
| #222 | A5/C5 | 88.24 | 3.47±0.07cde | 0.61±0.05b | 88.19±0.61a | 3.66±0.12d | 2.26±0.17d | 0.37±0.07a | 1.44±0.32ab | - |
| #230 | A5/C5 | 88.82 | 3.36±0.13de | 0.72±0.06b | 88.56±0.66a | 3.71±0.29d | 1.86±0.11d | 0.35±0.07a | 1.44±0.21ab | - |
| #238 | A5/C5 | 86.43 | 3.41±0.19cde | 0.53±0.06b | 86.41±1.09a | 4.23±0.27d | 3.52±0.46c | 0.34±0.08a | 1.56±0.37a | - |
| #240 | A5/C5 | 88.25 | 3.82±0.15c | 0.89±0.07ab | 88.02±0.58a | 3.92±0.21d | 1.93±0.13d | 0.47±0.06a | 0.95±0.13bc | - |
| #289 | A5/C5 | 88.45 | 3.27±0.12e | 0.56±0.06b | 88.65±0.27a | 3.67±0.13d | 2.14±0.15d | 0.32±0.07a | 1.39±0.24ab | - |
| #294 | A5 | 78.80 | 4.29±0.16b | 0.77±0.06b | 76.91±1.37b | 10.56±1.10c | 5.83±0.13b | 0.46±0.19a | 1.18±0.04abc | - |
| #314 | A5/C5 | 88.38 | 3.57±0.09cde | 0.83±0.08ab | 88.27±0.58a | 3.52±0.11d | 2.21±0.39d | 0.35±0.04a | 1.25±0.09abc | - |
