# Supplementary material for: CRISPR/Cas9-mediated editing of double loci of BnFAD2 increased the seed oleic acid content of rapeseed (Brassica napus L.)
Source: Front Plant Sci. 2022 Nov 22;13:1034215. doi: 10.3389/fpls.2022.1034215 (PMC9723152; doi:10.3389/fpls.2022.1034215)
Supplement: Supplementary file 2 [file DataSheet_2.docx]

Figure S1 The lowest and highest temperature from Oct to May in 2018, 2019, 2020, and 2021 with different generations of mutants from T1 to T4


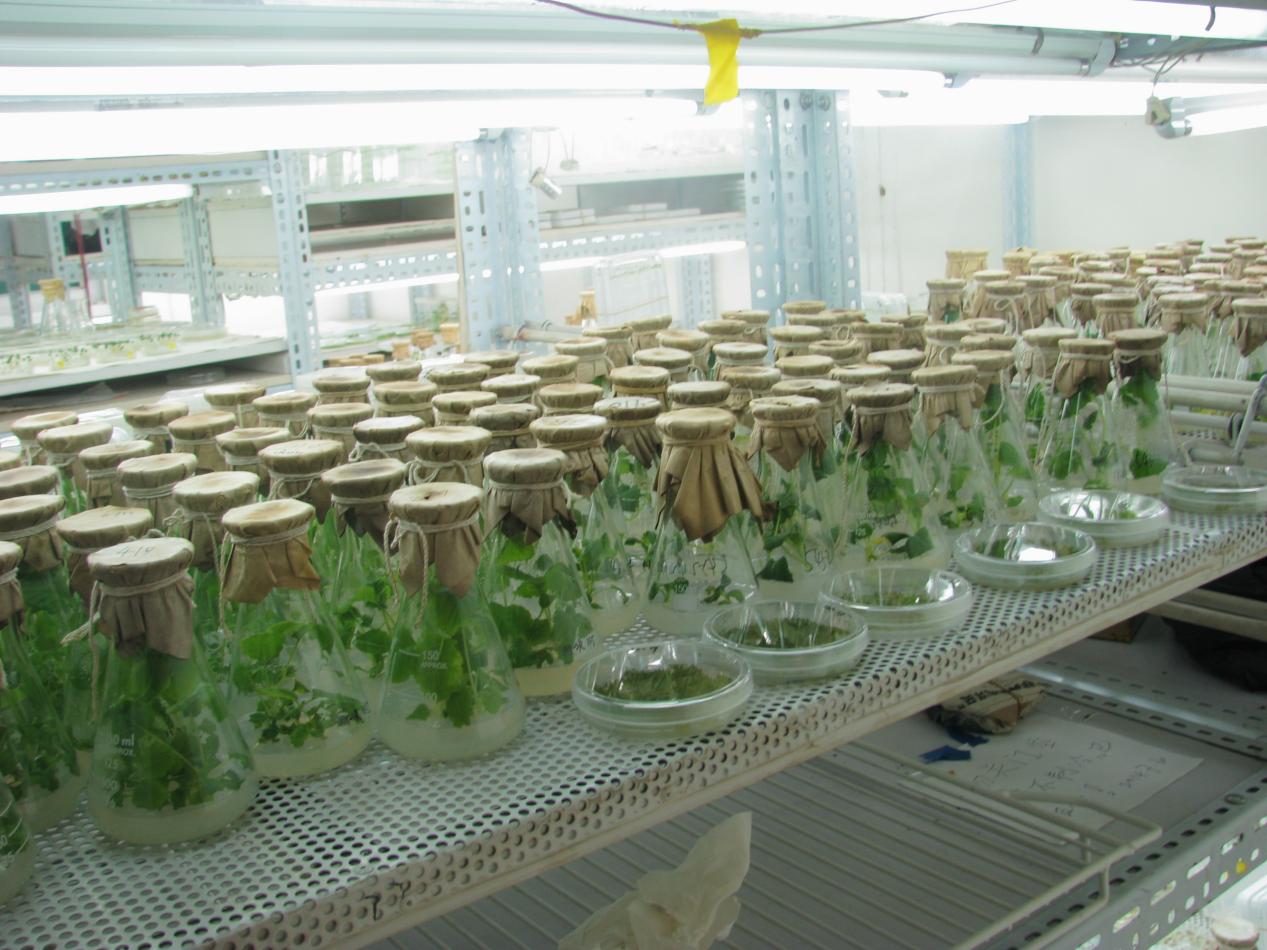


Figure S2 regeneration plants from B57-1


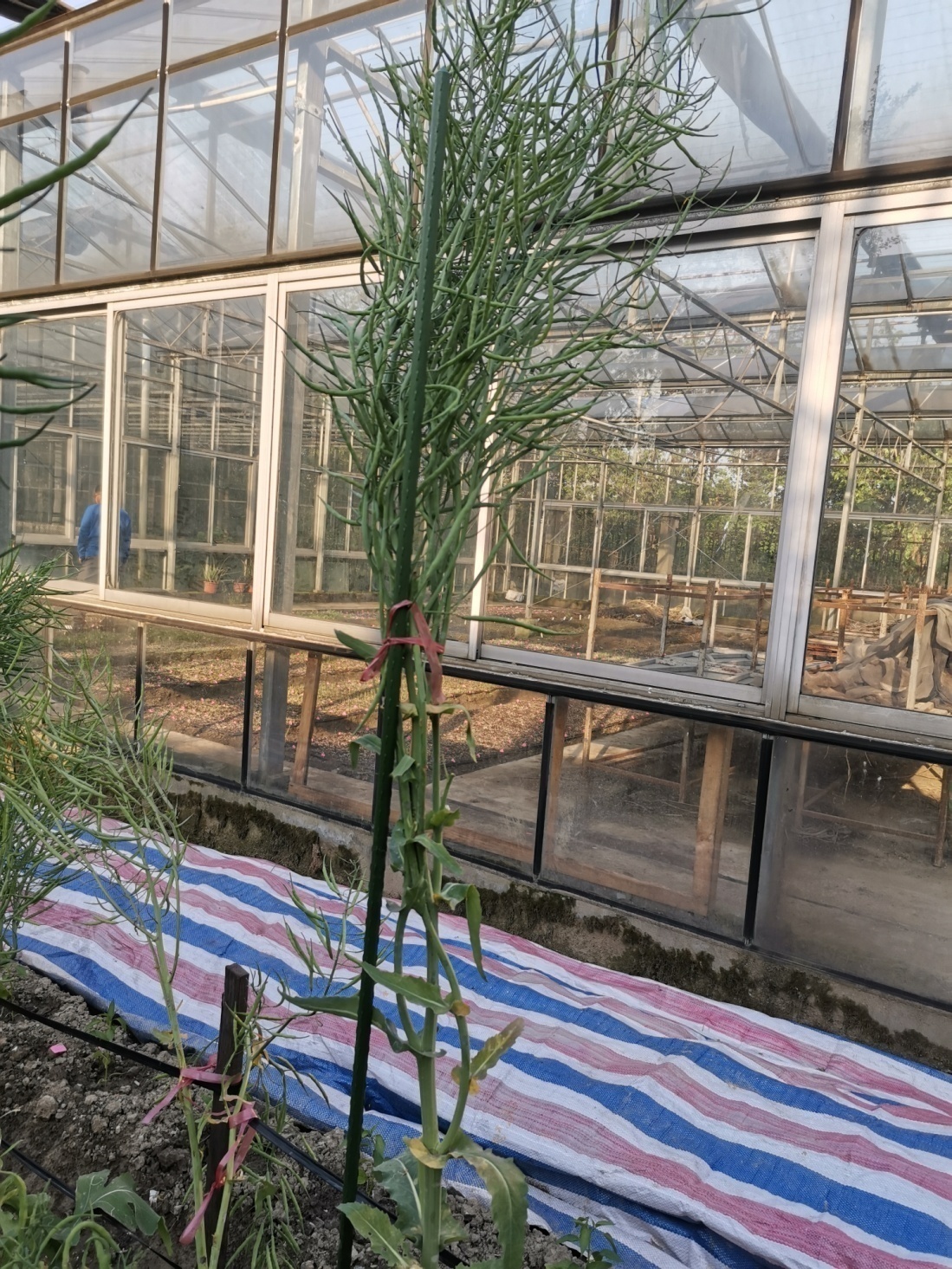


Figure S5 double loci edited plant (#289)
