# Supplementary figures and images for: CRISPR/Cas9-mediated editing of double loci of BnFAD2 increased the seed oleic acid content of rapeseed (Brassica napus L.)
Source: Front Plant Sci. 2022 Nov 22;13:1034215. doi: 10.3389/fpls.2022.1034215 (PMC9723152; doi:10.3389/fpls.2022.1034215)

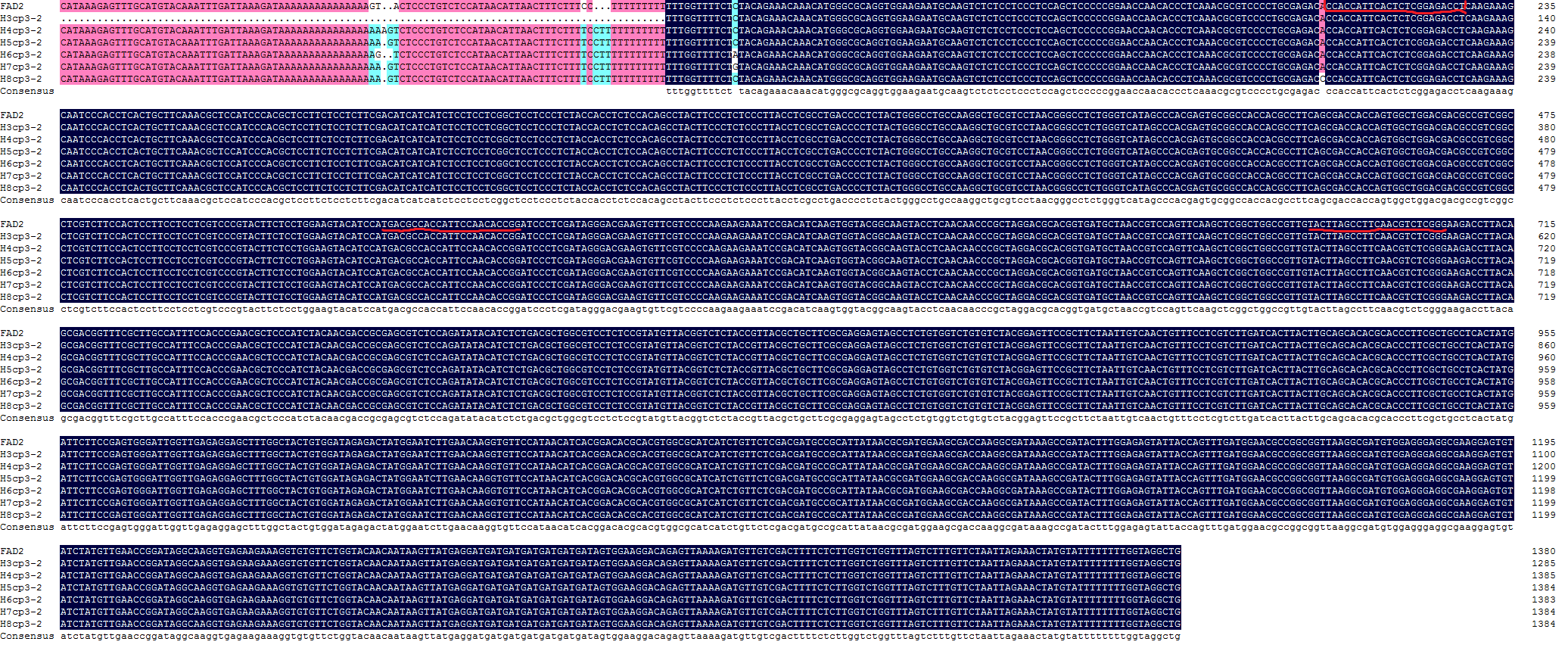

Supplement: Supplementary file 3 [file Image_1.tif]

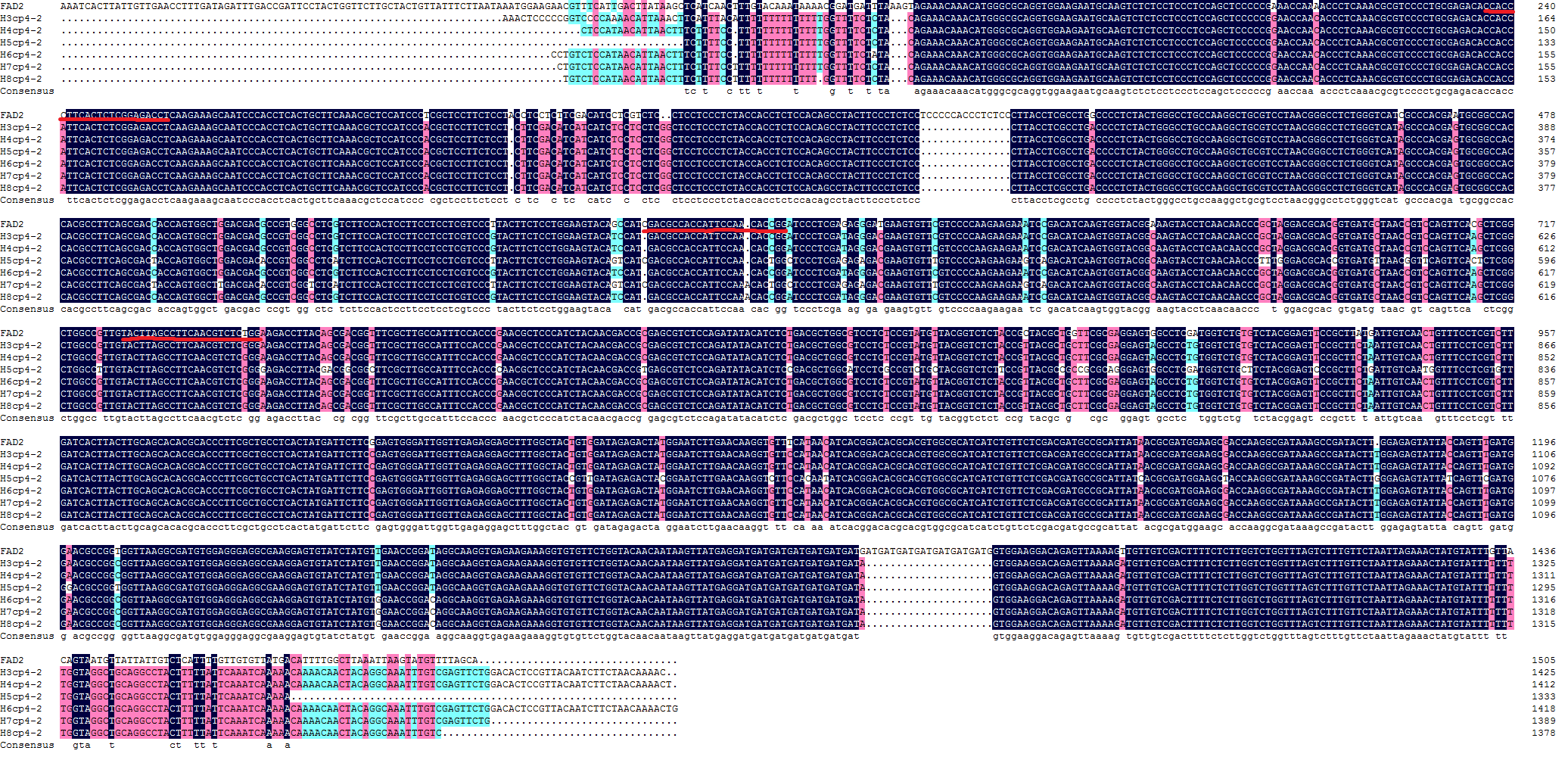

Supplement: Supplementary file 4 [file Image_2.tif]
